# Characterization of carbonated beverage fortified with chamomile herbal extract

**DOI:** 10.1002/fsn3.4101

**Published:** 2024-03-15

**Authors:** Muhammad Aamir, Ahtesham Abid, Iqra Azam, Ali Ikram, Farhan Saeed, Muhammad Afzaal, Huda Ateeq, Noor Akram, Shahzad Hussain, Mahbubur Rahman Khan

**Affiliations:** ^1^ National Institute of Food Science and Technology (NIFSAT) University of Agriculture Faisalabad Pakistan; ^2^ Department of Food Science and Technology Government College Women University Faisalabad Faisalabad Pakistan; ^3^ University Institute of Food Science and Technology The University of Lahore Lahore Pakistan; ^4^ Department of Food Science Government College University Faisalabad Faisalabad Pakistan; ^5^ Food Safety & Biotechnology Lab, Department of Food Science Government College University Faisalabad Faisalabad Pakistan; ^6^ Department of Food Science and Nutrition, College of Food and Agriculture King Saud University Riyadh Saudi Arabia; ^7^ Department of Food Processing and Preservation Hajee Mohammad Danesh Science & Technology University Dinajpur Bangladesh

**Keywords:** antioxidant, beverage, chamomile extract, fortification

## Abstract

The current study was planned to provide nutrient dense carbonated beverage fortified with chamomile herbal extract that was rich in healthy nutrients and best to use. Infusion method was used to prepare chamomile herbal extract. After adding flavor and sugar syrup, carbonation was done. Different treatments were prepared (T_0_, T_1_, T_2_, T_3_, T_4_, and T_5_). The antioxidant potential, physiochemical properties, and sensory attributes of beverage were assessed. Results showed that addition of chamomile enhanced the antioxidant and physiochemical properties of beverage. The DPPH activity, total phenolic content, and total flavonoid content were observed as 49.23 ± 0.03 (%), 136.92 ± 0.06 (mg GAE/L), and 1989.47 ± 0.07 (mg QE/L), respectively, among T_5_ with 12% of chamomile extract. Moreover, the acidity increases from T_0_ to T_5_ (0.191 ± 0.01 to 0.220 ± 0.01). Furthermore, the overall acceptability of T_4_ was highest among sensory attributes.

## Graphical abstract

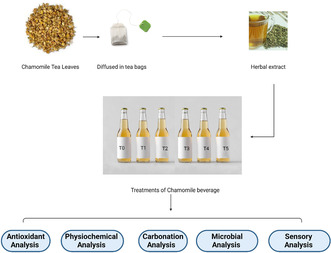

## 1 INTRODUCTION

Among the most ancient therapeutic plants, Chamomile is a participant of the Asteraceae species and is attributed by two assortments, *Chamomilla Recutita* (German) and *Chamaemelum Nobile* (Roman). The dried‐out parts of the chamomile herb consist of numerous terpenes and flavones, which may lead to its therapeutic qualities. Chamomile has historically been used as an anti‐inflammatory, slightly astringent, antioxidant, and comforting medication for decades (Perestrelo et al., 2022). For several human illnesses, such as hay fever, swelling, muscle pains, menstrual problems, sleeplessness, ulcers, fractures, hemorrhage, stomach trouble, and rheumatoid arthritis, chamomile preparations are mostly used. Essential chamomile oils are generally used in cosmetics and aromatherapy (Bravo et al., 2022). Chamomile has been prescribed for several healing purposes and is one of the earliest, most commonly cultivated, and well‐known therapeutic plants in the globe (Suna et al., 2019). Chamomile has been used externally to cure bacterial infection, scarlet fever, diseases of the ears and lungs, eye conditions involving tear ducts clogged, cellulitis, nose irritation, and ivy poison (Bayliak et al., 2021).

Chamomile contains a variety of active ingredients that have been extracted and used in therapeutic and cosmetic formulations. The plant contains 0.25%–1.90% essential oil, which is made up of many different oils. The oil varies in color from bright blue to dark green when raw, but turns heavy yellow upon storage when subjected to steam distillation. Regardless of the fact that it fades, the oil retains its effectiveness. Chamomile contains about 120.0 secondary metabolites, including 27 terpenoids and 35 flavonoids. The terpenoids bisabolol and its oxide azulenes, containing chamazulene and acetylene compounds, are the core elements of the essential oil isolated from German chamomile plants (Mosallanezhad et al., 2021).

Numerous phenolic chemicals, particularly the flavones apigenin, quercetin, and patuletin as glucosides, and other acetylated metabolites, are also prominent ingredients of the blossoms. Apigenin, the most potential flavonoid, is the most promising flavonoid (Catani et al., 2021). It can be found in trace amounts as pure apigenin; however, it is most commonly found in the shape of glycosides. Some of the flavones present in matricaria, such as apigenin, luteolin, and apian, as well as coumarins, and thiophene compounds, are also found in Roman chamomile (Yousefbeyk et al., 2022).

Therefore, chamomile is becoming an effective and excellent herb and is used in various forms, that is, as dry powders, oral infusions, tea preparation, etc. Many researchers suggest the utilization of chamomile flowers dry powder for a variety of traditional health concerns. Adults should take 2–3 g of dry flower head powder 2–4 times daily to acquire the stimulating efficacy (Karaaslan Ayhan, 2021). Chamomile tea is among the most famous herbal teas in the globe, with over a thousand cups sipped each day. Chamomile infusions and chamomile tea are the same thing. Because chamomile is sold in market as “chamomile tea packs,” the term “tea” has become increasingly widespread. Teabags including chamomile floral dried form, either pure or combined with other famous therapeutic herbs, are also commercially available (Sotiropoulou et al., 2020).

Chamomile tea only includes 10.0% of the essential oil components and roughly 29%–30.0% of the flavonoids. Dry petals are typically placed in a saucepan of hot water after being washed with ice water. The tea is obtained by covering the pot for 5–6 min; other herbs can be included for added nutritious/therapeutic benefit. To enhance the sweetness of tea, honey or sugar can be applied. Chamomile tea should always be made in a closed container to avoid steam from escaping, as evaporation reduces the therapeutic efficacy of the herbs significantly (Srivastava & Gupta, 2007). Caffeine, which is a xanthine alkaloid extracted from the beans, flowers, and fruit of herb, is one of the added extracts, in particular from coffee plants, tea, kola nuts, or cacao (Suna et al., 2019).

Various studies have evaluated the effect of herbal tea fortified with bioactive compounds, such as Hegde et al. (2022), in their study developed herbal tea from *Bacopa monnieri*. Similarly, Roy et al. (2022) in their study developed herbal formulation by using four herbal plants to determine anxiolytic efficacy in a rat model study. In another study by Tamer et al. (2021), green and black sweetened tea was used to develop kombucha beverage, as source of potential herbs, Linden, lemon balm, sage, echinacea, mint, and cinnamon were added to beverage. Realizing the significance of chamomile extract in diet, the present project is designed to provide a healthy and nutritious carbonated beverage fortified with chamomile herbal extract that will be rich in essential bioactive compounds as well as will make the best use of the herbal extract in daily diet.

## 2 MATERIALS AND METHODS

### 2.1 Procurement of raw materials

Chamomile tea leaves were acquired from the local market of Faisalabad. Rest of the raw materials, that is, sugar, stabilizers, and essence were purchased from the superstore of Faisalabad. The regents and chemicals used were from Merck, Germany. Moreover, lab facilities were availed at the National Institute of Food Science and Technology, UAF, Faisalabad, and for further analysis, the facilities at Food Safety & Biotechnology Lab, GCUF were availed.

### 2.2 Preparation of raw materials

Chamomile tea leaves were taken, cleaned, and sun dried, after drying powder was made of tea leaves by using lab grinder. The herbal powder was infused in tea bags of 5 g, later on, herbal tea bags were suspended in 100‐mL boiling water for 4–6 min, as directed by the technique by Horžić et al. (2009). After that, the extract was blended with sugar syrup, and the desired flavors were added. The beverage was filled in glass bottles and carbonation was done, beverage carbonation was achieved through the injection of CO_2_ into bottles at a pressure of 15 psi for 15 s, followed by prompt cooling to 5 °C, as previously done (Atallah & Gemiel, 2020).

### 2.3 Product development

Herbal beverage formulations were made by using different concentrations of chamomile extract and carbonation (Table 1).

**TABLE 1:** Treatment plan.

Treatment	Water (v/v%)	Chamomile extract (v/v%)	Sugar syrup (w/v%)
T_0_	100	0	0
T_1_	94	4	2
T_2_	92	6	2
T_3_	90	8	2
T_4_	88	10	2
T_5_	86	12	2

*Note*: T_0_ = water/chamomile extract/syrup ratio (100:0:0); T_1_ = water‐to‐chamomile extract ratio (94:4:2); T_2_ = water‐to‐chamomile extract ratio (92:6:2); T_3_ = water‐to‐chamomile extract ratio (90:8:2); T_4_ = water‐to‐chamomile extract ratio (88:10:2); T_5_ = water‐to‐chamomile extract ratio (86:12:2).

### 2.4 Antioxidant activity

#### 2.4.1 Total phenolic content

The total phenolic content (TPC) of the samples was measured by Folin–Ciocalteu method as followed by Várady et al. (2020). Folin–Ciocalteu reagent was combined in a flask along with 0.75 mL of saturated sodium carbonate solution and 0.95 mL of distilled water. The mixture was then incubated at 37 °C for 30 min, and the absorbance was measured at 765 nm with an ultraviolet–visible spectrophotometer (Unicam Heλio α Cambridge, UK). The results were compared to a gallic acid solution‐based standard curve (Sigma Chemical). Milligrams of gallic acid equivalents per gram of fresh weight were used to calculate the TPC (mg GAE/g FW). Total phenolic compounds of each extract in gallic acid equivalents were calculated by using the different formulas:

C=c×V/m,

where *C* is the total phenolic contents, *c* is the concentration of gallic acid, *V* is the volume of extract, and *m* is the weight of sample.

#### 2.4.2 Total flavonoid content

The total flavonoid content (TFC) was determined using the AlCl_3_ colorimetric technique as followed by Tristantini and Amalia (2019), to assess flavonoid content, the quercetin reagent was employed as a standard. The stock quercetin solution was made by dissolving 5 mg of quercetin in 1 mL of methanol. Several dilutions in the range of 5–200 μg/mL were also made. After that, 6 mL of sample or standard solution was combined with 6‐mL 2% solution of AlCl_3_. The solution was correctly mixed and incubated for roughly an hour at room temperature. The absorbance of the sample and standard was then measured using a spectrophotometer at 420 nm. The TFC was calculated using the following formula:

Y=0.0162x+0.0044.

TFC was expressed as quercetin equivalent mg/g.

#### 2.4.3 DPPH radical scavenging activity

The ability of the extract to react with the DPPH free radical was tested to determine its antioxidant capability (Chandrasekar et al., 2006). The sample was dissolved with 5 mL of 80% methanol and agitated for 2 h in a shaker. After this, 2‐mg DPPH was dissolved in 50‐mL methanol to make the DPPH solution. Then, 25 μL of methanol extract was added to 2 mL of this solution. To complete the reaction, the mixture was agitated and placed in a dark place. The absorbance value was found to be 515 nm. The DPPH radical scavenging % was calculated with the formula below:

$$\text{Radical scavenging activity}\;\left(\%\right)=1-A_{f}/A_{0}\times 100,$$

where *A*_0_ and *A*_f_ are the blank and sample absorbance values.

### 2.5 Physiochemical analysis

#### 2.5.1 Determination of pH

Each sample was measured for its pH with a digital pH meter after 30‐day period during 4 months of storing following the procedure of AOAC (2016).

#### 2.5.2 Total titratable acidity

Each sample was measured for its titratable acidity after 30‐day period during 4 months of storing following the procedure of AOAC (2016).

#### 2.5.3 Total soluble solids

Total soluble solids (TSS) of product were measured after 30‐day period during 4 months of storing following the procedure of AOAC (2016) by using refractometer.

### 2.6 Carbonation analysis

The carbonation tester was properly calibrated using CO_2_ gas standards. The beverage sample was allowed to reach the desired testing temperature. The pressure regulator of the container was attached to the container of the beverage sample. For the determination of carbonation level, the rest of the method was followed as previously done by Mandrah et al. (2017).

### 2.7 Microbial study

Total plate count (TPC) was recorded as a colony‐forming unit. Ten‐gram sample was mixed properly in 90 mL of saline solution and diluted serial wise up to 10^−6^ dilution. A 0.1 mL of sample from each serial dilution was placed on prepared general media plates under sterilized environment. After that, inoculated plates were incubated at 37°C for 24–48 h.

$$\begin{array}{c} \mathrm{TPC}\;\left(\mathrm{CFU}/\mathrm{mL}\right)=\text{Number of colonies}\times \text{dilution factor}/\\ \;\text{volume of sample taken}. \end{array}$$

### 2.8 Sensorial analysis of beverage

A panel of judges evaluated beverage samples for sensory features using a 9‐point Hedonic score system based on color, sweetness, bitterness, aroma, mouthfeel, appearance, and overall acceptability as prescribed by Lawless et al. (2010) and Jara‐Solis et al. (2023). The organoleptic features of beverage were analyzed by 20 students aged 20–30 years from University of Agriculture Faisalabad.

### 2.9 Statistical analysis

All the experiments were executed three times and the data were subjected to statistical analysis. The acquired data were subjected to SPPS, version 2.0 for variance analysis under Complete Randomized Design and Factorial Design for the evaluation of means square and mean analytical values using the Montgomery method.

## 3 RESULTS AND DISCUSSIONS

### 3.1 Antioxidant potential of beverage

Antioxidant activity of chamomile‐containing beverage was assessed by 2,2‐diphenyl‐1 picrylhydrazyl (DPPH). Table 2 indicates that the value of DPPH was affected significantly by the addition of chamomile extract, and the DPPH activity increased with the increase of chamomile extract throughout the treatments from T_0_ to T_5_. Table 2 presents the results in means ± SD, showing the range of variation in values from 41.09 ± 0.05 (%) to 49.23 ± 0.03 (%). Maximum range (49.23 ± 0.03) was observed in beverage with 12% chamomile herbal extract added. The minimum value (41.09 ± 0.05) was observed in the control with no addition of chamomile extract. The increase in antioxidant activity in the current study followed the same trend as reported by Suna et al. (2019), who determined the antioxidant activity in herbal tea beverage extract and rooibos extract. These results also aligned with the work done by Jakubczyk et al. (2020) who investigated the chemical profile and antioxidant activity of Kombucha tea. The author also reported the increasing trend in antioxidant activity of the beverage prepared from green tea.

**TABLE 2:** Phytochemical analysis of beverage.

Treatments	DPPH (%)	TPC (mg GAE/L)	TFC (mg QE/L)
T_0_	41.09 ± 0.05^b^	119.67 ± 0.02^a^	1825.99 ± 0.03^a^
T_1_	43.18 ± 0.09^a^	122.91 ± 0.05^b^	1857.88 ± 0.07^d^
T_2_	45.85 ± 0.11^c^	125.46 ± 0.01^c^	1891.34 ± 0.01^c^
T_3_	46.98 ± 0.14^f^	128.65 ± 0.07^d^	1933.09 ± 0.08^b^
T_4_	47.91 ± 0.06^e^	132.97 ± 0.03^e^	1956.99 ± 0.05^f^
T_5_	49.23 ± 0.03^d^	136.92 ± 0.06^f^	1989.47 ± 0.07^e^

*Note*: Results have been presented in means ± SD. T_0_ = water/chamomile extract/syrup ratio (100:0:0); T_1_ = water‐to‐chamomile extract ratio (94:4:2); T_2_ = water‐to‐chamomile extract ratio (92:6:2); T_3_ = water‐to‐chamomile extract ratio (90:8:2); T_4_ = water‐to‐chamomile extract ratio (88:10:2); T_5_ = water‐to‐chamomile extract ratio (86:12:2).

The different superscripts are denoting that one treatment and values for each treatment are significantly different from others.

The mean TPC values exhibited a range of variation from 119.67 ± 0.02 (mg GAE/L) to 136.92 ± 0.06 (mg GAE/L) as listed in Table 2. Notably, the highest range (136.92 ± 0.06) was observed in the beverage enriched with a 12% chamomile herbal extract, while the lowest value (119.67 ± 0.02 mg GAE/L) was noted in the control group without any addition of chamomile extract. This disparity underscores the impact of chamomile herbal extract on the TPC of the beverages. The findings align with a study conducted by Do et al. (2014), where alterations in TPCs of herbal tea extracts were investigated by introducing various solvents. Consistent with our results, Do et al. reported increasing trends in TPC values, reinforcing the validity and consistency of our observations. In a parallel exploration, Badejo et al. (2020) delved into the TPC of beverages derived from *Cyperus esculentus*. Their research documented higher TPC values ranging from 21.68 ± 0.58 **(**mg GAE/L**)** to 45.68 ± 2.07 **(**mg GAE/L**)**. Remarkably, these values closely parallel the TPC range observed in our current study, providing additional support and validation for the observed trends in phenolic content. Moreover, the present study's TPC findings, influenced by chamomile herbal extract addition, are consistent with existing literature, particularly the increasing trends noted in the presence of specific additives, as demonstrated by Do et al. (2014), and the comparable TPC values within the range reported by Badejo et al. (2020) in analogous beverage formulations.

The mean values for TFCs have been shown in Table 2, spanned a range from 1825.99 ± 0.03 (mg QE/L) to 1989.47 ± 0.07 (mg QE/L) in the tested beverages. Notably, the maximum TFC range (1989.47 ± 0.07) was observed in the beverage enriched with a 12% chamomile herbal extract, while the minimum value (1825.99 ± 0.03 mg QE/L) was recorded in the control group with no chamomile extract addition. This reveals the influence of chamomile herbal extract on the observed variation in TFC values. The ascending trend in TFC values following chamomile extract addition aligns with findings reported by Bustos et al. (2019). In their investigation involving fermented beverages and the plant *Parastrephia lucida*, significant variations in TFC values were noted. The TFC values in that study ranged from 333.47 ± 12.80 to 601.12 ± 0.04 (mg QE/L). This trend is further supported by the work of Chang et al. (2020), who explored the phytochemical effects of medicinal plant‐based concentrated beverages. In their study, an increasing trend in TFC was observed, with values ranging from 1.59 to 132.39 mg QE/g. This parallels our findings and provides additional corroboration for the impact of chamomile extract on elevating TFC values in beverage formulations. Furthermore, the current study demonstrates a consistent increase in TFC values upon chamomile herbal extract addition, aligning with similar trends observed in studies by Bustos et al. (2019) and Chang et al. (2020). These findings contribute to the growing body of evidence supporting the positive influence of specific herbal extracts on the flavonoid content of beverages.

### 3.2 Physiochemical profile of beverage

#### 3.2.1 pH

The means of pH have been listed in Table 3, results showed that the pH means exhibited a range from 5.35 ± 0.01 to 3.34 ± 0.04 in the beverages under investigation. Remarkably, the lowest pH value (3.34 ± 0.04) was observed in the beverage containing a 12% chamomile herbal extract, while the highest pH value was recorded in the control group without any chamomile extract addition. This indicates that the inclusion of chamomile extract led to a decrease in pH, resulting in increased acidity in the beverage formulations.

**TABLE 3:** Physiochemical profile of beverage.

Treatments	Titratable acidity	pH	TSS	Carbonation level
T_0_	0.191 ± 0.01^e^	5.35 ± 0.01^a^	12.81 ± 0.03^f^	3.2 ± 0.01^e^
T_1_	0.195 ± 0.00^b^	4.99 ± 0.10^b^	12.83 ± 0.08^e^	3.19 ± 0.10^d^
T_2_	0.201 ± 0.00^f^	4.65 ± 0.15^c^	12.84 ± 0.09^d^	3.18 ± 0.15^a^
T_3_	0.206 ± 0.02^d^	4.41 ± 0.11^d^	12.86 ± 0.013^c^	3.17 ± 0.11^d^
T_4_	0.210 ± 0.01^a^	3.65 ± 0.06^e^	12.87 ± 0.05^b^	3.15 ± 0.06^b^
T_5_	0.220 ± 0.01^c^	3.34 ± 0.04^f^	12.89 ± 0.07^a^	3.14 ± 0.04^c^

*Note*: Results have been presented in means ± SD. T_0_ = water/chamomile extract/syrup ratio (100:0:0); T_1_ = water‐to‐chamomile extract ratio (94:4:2); T_2_ = water‐to‐chamomile extract ratio (92:6:2); T_3_ = water‐to‐chamomile extract ratio (90:8:2); T_4_ = water‐to‐chamomile extract ratio (88:10:2); T_5_ = water‐to‐chamomile extract ratio (86:12:2).

The different superscripts are denoting that one treatment and values for each treatment are significantly different from others.

These findings are consistent with those reported by Jakubczyk et al. (2020), who studied kombucha beverages prepared from green tea, black tea, and red tea. Their research demonstrated a decreasing trend in pH values over time during the preparation of beverages from green tea extract, aligning with the observed impact of chamomile extract on pH in our study. In a parallel study, Atallah and Gemiel (2020) explored the effect of herbal extract addition on pH values while characterizing carbonated beverages with the incorporation of herbal extract and fruit juice. Their findings revealed a significant decrease in pH values from 6.37 to 3.89 upon the addition of herbal extract. This consistent trend supports our observations regarding the decrease in pH with chamomile extract addition.

Moreover, the addition of chamomile extract to the beverages resulted in a notable decrease in pH, aligning with similar trends observed in studies by Jakubczyk et al. (2020) and Atallah and Gemiel (2020). These findings underscore the impact of herbal extracts on the acidity of beverages and contribute valuable insights to the characterization of such formulations.

#### 3.2.2 Titratable acidity

Table 3 presents the means of titratable acidity, ranging from 0.191 ± 0.01 to 0.220 ± 0.01, across the tested beverages. Notably, the highest titratable acidity range (0.220 ± 0.01) was observed in the beverage enriched with a 12% chamomile herbal extract, while the lowest value (0.191 ± 0.01) was noted in the control group without any chamomile extract addition. This indicates that the addition of chamomile extract resulted in an increase in the titratable acidity of the beverages.

These findings are consistent with a study by Kaur et al. (2018), where the optimization of a beverage made from herbal extracts of sugarcane and radish revealed a significant impact of herbal extract addition on titratable acidity. The titratable acidity values in their study ranged from 0.7% to 1.3%, reinforcing the notion that herbal extracts can influence the acidity profile of beverages.

Additionally, Atallah and Gemiel (2020) reported variations in titratable acidity values from 0.1 to 0.3 as they introduced herbal extract (mint) along with other essential ingredients. Their study highlighted the significant effect of herbal extract on titratable acidity, further supporting our observations regarding the impact of chamomile extract on the acidity levels in beverages.

In summary, the addition of chamomile extract led to an increase in titratable acidity, aligning with similar trends observed in studies by Kaur et al. (2018) and Atallah and Gemiel (2020). These results contribute valuable insights into the titratable acidity modulation in beverages through the incorporation of herbal extracts.

#### 3.2.3 Total soluble solids

The findings of the study reveal a significant impact of chamomile extract addition on the TSS in the herbal beverage as shown in Table 3. The mean TSS values exhibited a range of variation, ranging from 12.81 ± 0.03 to 12.89 ± 0.07. According to Table 3, the lowest TSS value (12.81 ± 0.03) was noted in the beverage without added chamomile herbal extract, while the highest TSS value (12.89 ± 0.07) was observed in the beverage containing 12% chamomile herbal extract. This indicates a slight increase in TSS values with the addition of chamomile extract to the beverage. Notably, treatments with the highest chamomile extract concentration were found to be superior to the control.

The rising trend in TSS values suggests the occurrence of sedimentation in the herbal beverage, particularly in cases with significantly elevated TSS values. However, this marginal increase in TSS did not have an adverse effect on the sensory attributes of the beverage. These findings align with a previous study by Gimhani and Liyanage (2018), who also observed a similar increasing trend in TSS values in a ready‐to‐use beverage.

Furthermore, the research conducted by Balaji and Prasad (2014), investigating the addition of herb cardamom and ginger to a beverage, reported a similar upward trend in TSS values, corroborating the outcomes of the present study. These consistent findings across different studies underscore the reliability of the observed effects of herbal extract additions on TSS values in beverages.

#### 3.2.4 Carbonation level

The findings of the study demonstrate a significant impact of chamomile extract addition on the carbonation level in the beverage. The mean carbonation levels exhibited a range of values from 3.20 ± 0.01 to 3.14 ± 0.04. The lowest carbonation value (3.14 ± 0.04) was observed in the beverage containing 12% chamomile herbal extract, while the highest carbonation value (3.20 ± 0.01) was noted in the control group without chamomile extract addition.

This indicates that the inclusion of chamomile extract in the beverage led to a decrease in carbonation value. These results align with the research conducted by Siddiqui et al. (2014), who investigated the impact of carbonation on a health drink. The observed trend in carbonation levels upon the addition of chamomile extract is consistent with the findings of Siddiqui et al., highlighting a similar effect on carbonation levels with the introduction of ingredients.

The parallel trends observed in carbonation levels in both studies suggest a reproducible influence of ingredient addition, specifically chamomile extract, on the carbonation characteristics of the beverage. This adds credibility to the observed decrease in carbonation values associated with chamomile extract supplementation in the present research.

### 3.3 Sensory analysis

#### 3.3.1 Color

It is depicted in Table 4 that mean value of color ranged from 5.11 to 8.10. The maximum score (8.10) has been given to T_1_ having 4% supplementation of chamomile herbal extract while minimum score (5.11) was given to T_5_. The trend of score given by panel of judges is T_1_ (8.10 ± 0.71) > T_2_ (7.32 ± 0.55) > T_0_ (7.30 ± 0.207) > T_3_ (6.80 ± 0.50) > T_4_ (6.610 ± 0.06) > T_5_ (5.11 ± 0.04). The panel found that the beverage made with 4% chamomile herbal extract supplementation was quite attractive while they slightly disliked the T_5_. At 12%, the color of the herbal beverage was relatively dark and, therefore, less acceptable. This showed that increase in quantity of chamomile herbal extract alters the taste of herbal beverage in an unacceptable manner. Suna et al. (2019) also reported a similar decreasing trend in color upon addition of chamomile herbal extract in beverage from 0% to 10%. He reported that above 10% supplementation turns beverage darker and less acceptable.

**TABLE 4:** Sensorial evaluation of chamomile herbal beverage.

Treatments	Color	Sweetness	Bitterness	Aroma	Mouthfeel	Appearance	Overall acceptability
T_0_	7.30 ± 0.27^b^	7.43 ± 0.01^a^	6.02 ± 0.01^e^	7.11 ± 0.01^e^	7.60 ± 0.01^e^	7.04 ± 0.01^e^	7.54 ± 0.01^e^
T_1_	8.10 ± 0.71^a^	8.11 ± 0.01^b^	7.09 ± 0.10^d^	8.39 ± 0.10^d^	8.23 ± 0.10^d^	8.38 ± 0.10^d^	8.51 ± 0.10^d^
T_2_	7.32 ± 0.55^c^	7.02 ± 0.01^c^	5.18 ± 0.15^a^	7.01 ± 0.15^a^	7.18 ± 0.15^a^	7.47 ± 0.15^a^	7.10 ± 0.15^a^
T_3_	6.80 ± 0.50^d^	6.13 ± 0.01^d^	6.01 ± 0.11^d^	5.56 ± 0.11^d^	6.09 ± 0.11^d^	5.89 ± 0.11^d^	5.97 ± 0.11^d^
T_4_	6.10 ± 0.06^f^	6.94 ± 0.02^e^	5.75 ± 0.06^b^	6.15 ± 0.06^b^	6.45 ± 0.06^b^	6.55 ± 0.06^b^	6.51 ± 0.06^b^
T_5_	5.11 ± 0.04^e^	5.10 ± 0.04^c^	5.04 ± 0.04^c^	5.64 ± 0.04^c^	4.94 ± 0.04^c^	5.31 ± 0.04^c^	5.25 ± 0.04^c^

*Note*: Results have been presented in means ± SD. T_0_ = water/chamomile extract/syrup ratio (100:0:0); T_1_ = water‐to‐chamomile extract ratio (94:4:2); T_2_ = water‐to‐chamomile extract ratio (92:6:2); T_3_ = water‐to‐chamomile extract ratio (90:8:2); T_4_ = water‐to‐chamomile extract ratio (88:10:2); T_5_ = water‐to‐chamomile extract ratio (86:12:2).

The different superscripts are denoting that one treatment and values for each treatment are significantly different from others.

#### 3.3.2 Sweetness

The mean square of scores were given to sweetness evaluation of different chamomile herbal beverage formulations. The results revealed that there was a significant variation in sweetness score for beverage among different formulations. Table 4 shows that mean value of sweetness of beverage ranged from 8.11 to 5.10. The treatment (T_1_) got maximum score (8.11) followed by T_0_, T_2_, T_4_, T_3_, and T_5_ with 7.43, 7.02, 6.94, 6.13, and 5.10 scores, respectively. Panel did not like the sweetness level of T_5_ while T_3_ and T_4_ were acceptable. This showed that increase in quantity of chamomile herbal extract alters the taste of herbal beverage in an unacceptable manner. The trend of score given by panel of judges is T_1_ > T_0_ > T_2_ > T_4_ > T_3_ > T_5_. The panel found that the beverage made with 4% chamomile herbal extract supplementation was quite attractive while they slightly disliked the T_5_. Balaji and Prasad (2014) reported a significant variable trend in sweetness of beverage on adding herbal extract‐supplemented herbal beverage.

#### 3.3.3 Bitterness

The results depicted that there appeared to be a significant variation in the score of bitterness for beverage among different treatments. The results showed that the mean value of bitterness ranged from 7.09 to 5.02 (Table 4). The maximum score (7.09) was given to T_1_ having 4% supplementation of chamomile herbal extract, while minimum score (5.02) was given to T_5_. T_1_ got a score of 7.09 while control beverage treatment T_0_ got 6.02. Judges did not like the taste of T_5_ that is why they gave the lowest score 5.02. This showed that increase in quantity of chamomile herbal extract alters the taste of herbal beverage in an unacceptable manner. The result of bitterness is supported by the trend observed by Kaur et al. (2018) who reported a decrease in taste score of beverage when supplementation of herbal extract in beverage increased from 0% to 10%.

#### 3.3.4 Aroma

The results depicted that the mean value of aroma of beverage ranged from 8.39 to 5.64. Maximum score (8.39) was given to T_1_ having 4% supplementation of chamomile herbal extract, while minimum score (5.64) was given to T_5_. T_1_ got a score of 8.39 while control herbal beverage treatment T_0_ got 7.11. Judges did not like the taste of T_5_ that is why they gave the lowest score 5.64. The scores for T_2_, T_3_, and T_4_ are 7.01, 5.56, and 6.15, respectively. This showed that increase in quantity of chamomile herbal extract influences the taste of herbal beverage in an unacceptable manner. The trend of score given by panel of judges is T_1_ > T_0_ > T_2_ > T_4_ > T_3_ > T_5_. The panel found that the beverage made with 2% chamomile herbal extract supplementation was quite attractive while they slightly disliked the T_5_. Vázquez‐Cabral et al. (2014) studied the same trend of aroma attribute in the herbal beverage obtained with the addition of herbal extract.

#### 3.3.5 Mouthfeel

The results showed that the mean square of scores given to mouthfeel of chamomile herbal beverage prepared from different formulations of herbal extract concentration ranged from 8.23 to 4.94. The results depicted that there appeared to be a significant variation in the score of mouthfeel for beverage among different treatments.

The maximum score was given to T_1_ having 4% supplementation of chamomile herbal extract, while the minimum score was given to T_5_. T_1_ got a score of 8.23 while control herbal beverage treatment T_0_ got 4.94. Judges did not like the taste of T_5_ that is why they gave the lowest score 5.02. This showed that increase in quantity of chamomile herbal extract alters the taste of herbal beverage in an unacceptable manner. The trend of score given by panel of judges is T_1_ > T_0_ > T_2_ > T_4_ > T_3_ > T_5_. The panel found that the beverage made with 4% chamomile herbal extract supplementation was quite attractive while they slightly disliked the T_5_. At 10%, the bitterness hides the sweetening factor of beverage.

Chang et al. (2020) studied the increasing trend in mouthfeel effect of herbal extract addition in medicinal plant‐based medical concentrated beverages. The range of values was from 7.59 to 4.39. It showed the same trend as that of the present research.

#### 3.3.6 Appearance

It has been depicted that supplementation has significantly affected the texture. The results revealed that means of appearance have ranged from 8.38 to 5.31. A decrease in the appearance has been seen with an increase in the level of herbal extract. The trend of score given by panel of judges is T_1_ > T_0_ > T_2_ > T_4_ > T_3_ > T_5_.

The panel found that the beverage made with 2% chamomile herbal extract supplementation was quite attractive while they slightly disliked the T_5_. The maximum score (8.38) has been given to T_1_ herbal beverage followed by 4% supplemented herbal extract while the minimum score (5.31) has been given to 10% supplemented herbal beverage extract. Hence, an increase quantity of chamomile herbal extract affected the overall appearance of the beverage. Contreras‐López et al. (2021) reported the same trend of effect of herbal extract addition in beverage 6.85–4.55 when supplementation of chamomile herbal extract increased from 0% to 10%.

#### 3.3.7 Overall acceptability

The results depicted that there is a significant variation in the score of overall acceptability for beverage among different treatments. The mean values of overall acceptability ranged from 8.51 to 5.25. The maximum score (8.51) was given to T_1_ having 4% supplementation of chamomile herbal extract, while minimum score (5.25) was given to T_5_. T_1_ got a score of 8.51 while control herbal beverage treatment T_0_ got 7.54. Judges did not like the taste of T_5_ that is why they gave the lowest score 5.25. This showed that increase in quantity of chamomile herbal extract affects the overall acceptability of herbal beverage in an acceptable manner.

The trend of score given by panel of judges is T_1_ > T_0_ > T_2_ > T_4_ > T_3_ > T_5_. The panel found that the beverage made with 4% chamomile herbal extract supplementation was quite attractive while they slightly disliked the T_5_. The T_1_ was proved as the best accepted treatment with acceptable color, taste, aroma, etc. Atallah and Gemiel (2020) studied the significant effect of the addition of herbal extract on the overall acceptability value while characterizing the carbonated beverage by the addition of herbal extract and fruit juice. They reported a decreasing trend in overall acceptability on the addition of herbal extract from 6.38 to 4.09. This study supported the same trend of variation as in the current study.

## 4 CONCLUSION

The present study concluded that the addition of chamomile significantly improved the antioxidant capacity and physiochemical characteristics of the beverage. Specifically, the beverage with 12% chamomile extract (T_5_) exhibited notable antioxidant activity, with DPPH activity recorded at 49.23 ± 0.03%, TPC at 136.92 ± 0.06 mg GAE/L, and TFC at 1989.47 ± 0.07 mg QE/L. Additionally, the acidity levels increased progressively from T_0_ to T_5_, ranging from 0.191 ± 0.01 to 0.220 ± 0.01. Notably, T_4_, with an intermediate chamomile concentration, achieved the highest overall acceptability among sensory attributes. These findings underscore the potential of chamomile extract to enhance both the nutritional profile and sensory appeal of carbonated beverages, providing a valuable contribution to the development of health‐promoting and enjoyable beverage options.

## AUTHOR CONTRIBUTIONS

Ahtesham Abid and Iqra Azam designed the study and conducted under the supervision of Muhammad Aamir and Muhammad Afzaal. Ahtesham Abid, Ali Ikram, Huda Ateeq, and Noor Akram performed the study and participated in drafting the article. Farhan Saeed helped in developing the whole concept and editing. Noor Akram and Ali Ikram helped in preparing figures and tables, the overall quality of the manuscript was maintained by Shahzad Hussain and Muhammad Afzaal wrote, edited, and revised the manuscript critically. Mahbubur Rahman Khan revised the final written paper. The final version of the manuscript has been read and approved by all listed authors.

## CONFLICT OF INTEREST STATEMENT

The authors declare no conflict of interest.

## Data Availability

Even though adequate data have been given in the form of tables and figures; however, all authors declare that if more data are required then the data will be provided on a request basis.
